# Gender Differences in Vitamin D Status and Determinants of Vitamin D Insufficiency in Patients with Chronic Obstructive Pulmonary Disease

**DOI:** 10.3390/nu15020426

**Published:** 2023-01-13

**Authors:** Maria Minter, Hanna Augustin, Jenny van Odijk, Lowie E. G. W. Vanfleteren

**Affiliations:** 1 Department of Lung Medicine, Angered Hospital, SV Hospital Group, 424 22 Angered, Sweden; 2 Department of Internal Medicine and Clinical Nutrition, Institute of Medicine, Sahlgrenska Academy, University of Gothenburg, 405 30 Gothenburg, Sweden; 3 Department of Respiratory Medicine and Allergology, Sahlgrenska University Hospital, 413 45 Gothenburg, Sweden; 4 COPD Center, Department of Respiratory Medicine and Allergology, Sahlgrenska University Hospital, 413 45 Gothenburg, Sweden

**Keywords:** COPD, 25-Hydroxyvitamin D, 25(OH)D, vitamin D insufficiency, gender, supplementation

## Abstract

Low vitamin D levels are common in Chronic Obstructive Pulmonary Disease (COPD) and have been associated with various adverse COPD-related outcomes. Recent data on vitamin D status in representative COPD cohorts in Scandinavia is lacking. This study aimed to assess vitamin D status and determinants of vitamin D insufficiency in patients with COPD who were attending a specialist secondary care COPD clinic in Southwestern Sweden. All patients who visited the COPD clinic for their first medical visit during two periods, 2017–2018 and 2021, were included in this observational study. Measurements of 25-Hydroxyvitamin D (25(OH)D), clinical data and documentation of supplements containing vitamin D were collected retrospectively from patients’ medical records. Multivariable logistic regression analysis was performed to identify determinants of the primary outcome, vitamin D insufficiency (25(OH)D < 50 nmol/L). A total of 667 patients were included, and 33% had vitamin D insufficiency. The median 25(OH)D was 62 nmol/L (43.5–83.1 nmol/L). Vitamin D insufficiency was related to the male gender, current smoking habits, a lack of supplements containing vitamin D and the winter season for blood sampling. In conclusion, vitamin D insufficiency is common in patients with COPD. Men had significantly lower levels of vitamin D but took vitamin D-containing supplements less frequently compared to women. Our findings can help clinicians to identify patients who are at risk of vitamin D insufficiency and allow correction with supplementation where appropriate.

## 1. Introduction

Chronic obstructive pulmonary disease (COPD) is a common disease that is characterized by persistent respiratory symptoms and airflow limitations [1]. COPD is a life limiting condition and a major cause of morbidity and mortality worldwide [1,2]. Although primarily a lung disease, COPD is considered a complex and heterogeneous disease with multiple extrapulmonary manifestations [3,4]. Malnutrition, muscle weakness and osteoporosis are examples of comorbidities often described in COPD [4,5,6,7]. Vitamin D deficiency and insufficiency (assessed as 25-Hydroxyvitamin D, 25(OH)D, in serum or plasma) are also more prevalent in patients with COPD compared to controls [8]. Both European [9,10] and Scandinavian [11,12,13] studies have found that low vitamin D levels are common in patients with COPD. The prevalence of 25(OH)D levels below 50 nmol/L varies between 21–77%, depending on the study [12,13,14,15]. In a Swedish cross-sectional study from 2014, Måhlin et al. found that 54% of patients with COPD, from outpatient clinics, had 25(OH)D levels below 50 nmol/L. The mean 25(OH)D level in the cohort was 51.5 nmol/L [13]. More recent data on vitamin D status in representative cohorts of patients with COPD in Scandinavia is lacking.

Although vitamin D is mainly known for its role in skeletal health, there is growing interest in its immunomodulatory effects [16]. Vitamin D deficiency has been implicated as a risk factor for development of airflow obstructions [17] and has also been related to lower lung function [18] and respiratory infections [19,20], including COVID-19 [21]. In patients with COPD, vitamin D deficiency (variously defined) has been associated with lower lung function, disease severity and exacerbations [12,14,15,22]. Low vitamin D status in patients with COPD can be linked to low sunlight exposure due to reduced outdoor activity, low-quality dietary intake, and skin aging [12,23].

Vitamin D is synthesized in the skin under the influence of ultraviolet B (UVB) radiation but can also be obtained from foods and supplements [24]. The vitamin D requirement can be covered by sun exposure, but during winter the sun exposure is insufficient under Nordic conditions [25]. Thus, those with limited sun exposure and limited intake of vitamin D are at risk of vitamin D deficiency or insufficiency [24,25]. 

Since vitamin D deficiency and insufficiency has been associated with various adverse COPD-related outcomes, we believe it is important to update the knowledge about vitamin D status in a representative cohort of patients with COPD in Sweden. In this study we therefor aimed to assess vitamin D status and determinants of vitamin D insufficiency in patients with COPD attending a specialized secondary care COPD clinic in Southwestern Sweden.

## 2. Materials and Methods

### 2.1. Enrollment and Data Collection

This study was an observational study. All patients visiting the specialized secondary care COPD clinic at Sahlgrenska University Hospital in Gothenburg, Sweden, for their first medical visit during two periods, January 2017 to December 2018 and January to December 2021, were included. During these periods, 744 first medical visits took place. The inclusion criteria were patients diagnosed with COPD, according to the Global Initiative for Chronic Obstructive Lung Disease (GOLD) criteria, with a forced expiratory volume in one second (FEV_1_)/forced vital capacity (FVC) of less than 0.70 [1]. Exclusion criteria were no measure of the primary outcome 25(OH)D in serum or plasma in connection with the visit. 25-Hydroxyvitamin D is routinely measured on patients’ first visit to the specialized secondary care COPD clinic. Clinical data were collected retrospectively from patients’ medical records.

Patient data obtained were vitamin D status, age, gender, body mass index (BMI) kg/m^2^, smoking status (current smokers, former smokers, or never smoked), nutrition screening score and documentation of any form of supplements containing vitamin D, including both prescribed drugs and oral nutrition supplements. BMIs were categorized as underweight <18.5 kg/m^2^, normal weight 18.5–24.9 kgm^2^, overweight 25–29.9 kg/m^2^ and obese >30 kg/m^2^, in accordance with the World Health Organization’s (WHO) definition [26]. Nutrition screening score was based on the result of a malnutrition screening tool developed for patients with COPD, including three questions about appetite, weight his-tory and BMI. The results are scored from 0–4 points. A score below 2 indicates no risk of malnutrition, whereas 2 and above indicate risk of malnutrition. [27]. 

Data related to the disease included lung function, measured as FEV_1_ in percent of predicted value (FEV_1_ % predicted), GOLD grade and group as 1–4 and A–D respectively [1] and COPD assessment test (CAT) score [28]. GOLD grade 1–4 are classifications of airflow limitation severity: GOLD 1 (mild) FEV_1_ ≥ 80% predicted, GOLD 2 (moderate) 50% FEV_1_ < 80% predicted, GOLD 3 (severe) 30% ≤ FEV_1_ < 50% predicted and GOLD 4 (very severe) FEV_1_ < 30% predicted. GOLD group A-D are based on assessment of patients’ symptoms and exacerbation history [1]. Lung function was measured with a spirometer, Master Screen Body from Jaeger with SentrySuite software using Hedenström as reference [29,30]. CAT is a health status questionnaire designed to assess the impact of COPD on a person’s life. Scores range from 0–40, where high scores represent poor health status [31]. GOLD uses CAT< or ≥10 as cut-off point [1]. In those cases where GOLD grade was not documented, classification was done retrospectively according to GOLD criteria [1].

Analysis of 25(OH)D was performed at the laboratory of The Department of Clinical Chemistry, Sahlgrenska university hospital. During 2017–2018, 25(OH)D was analysed in serum using the direct Competitive Chemiluminescence Microparticle Immunoassay method by the instrument Rocha Cobas. During 2021, 25(OH)D was analysed in plasma using Chemiluminescent Microparticle Immunoassay method by the instrument Abbot Alinity. Vitamin D status, the primary outcome, (25(OH)D) was categorized as deficient <25 nmol/L), insufficient <50 nmol/L and sufficient (25(OH)D ≥50 nmol/L as suggested by the Institute of medicine (IOM) [32]. Month of blood sampling was also collected.

### 2.2. Statistical Analyses

Statistical analyses were performed using IBM SPSS Statistics for Windows, version 28 (IBM Corp., Armonk, NY, USA). Statistical significance was set at *p* < 0.05 for all comparisons. All variables were tested for normality distribution using graphical plots. For continuous variables, medians and first and third quartiles are presented, as most data was not normally distributed. Categorical variables are presented as numbers and percentages. Pearson Chi-squared test and Fisher-Freeman-Halton Exact test were used to compare categorical variables between groups. Independent samples Mann Whitney U test was used to compare median value for continuous variables between two groups. 

Multivariable logistic regression analysis was used to identify determinants of the primary outcome, vitamin D insufficiency (25(OH)D ≥50 nmol/L coded as 0, and 25(OH)D <50 nmol/L coded as 1). Covariates were identified based on clinical relevance and previous studies [15,33]. Identified covariates included age, gender, BMI, FEV_1_ % predicted, CAT score, smoking status, supplementation containing vitamin D and season for 25(OH)D blood sampling. Collinearity between the potential determinants was controlled and CAT score was therefore excluded due to collinearity with FEV_1_ % predicted. Covariates included in the model were, age, gender (female or male), BMI, FEV_1_ % predicted, smoking (former/never or current), supplementation containing vitamin D (yes or no) and season for 25(OH)D blood sampling (summer or winter). Seasons was defined as summer season (May–October) and winter season (November–April).

## 3. Results

### 3.1. Patient Characteristics

**Figure 1:**
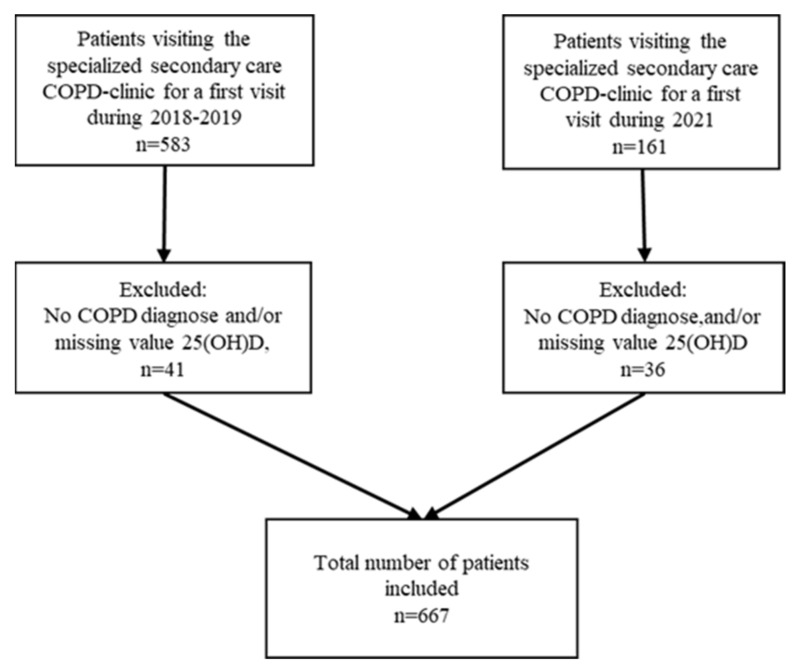
Flow chart of the inclusion in the study. COPD, Chronic obstructive pulmonary disease. 25(OH)D, 25-Hydroxy vitamin D.

In total, 667 patients were included in the study (Figure 1). Seventy-seven patients were excluded since they did not meet the criteria for COPD diagnosis or were not measured for 25(OH)D). For *n* = 35 of these patients, a current value for (FEV_1_ % predicted was missing since they could not perform a spirometry test at that first visit).

Patient characteristics are presented in Table 1. The median age was 73 years, ranging from 39–95 years. A total of 41% of patients were male, and almost one-third of the cohort was still smoking. Moderate to severe COPD (GOLD grade 2–3) was present in 77% of participants, and 15% had very severe COPD (GOLD grade 4). The median FEV_1_ % predicted was 47 (36–62). About half of the group (51%) reported high CAT scores, >20, indicating poor health status. Only 9% reported low CAT scores <10, indicating fewer symptoms and low health impact. About half of the group, 55%, had a BMI categorized as overweight or obese, and 8% were categorized as being underweight. Among participants that had been screened for risk of malnutrition, 34% had a score above 2, indicating a risk of malnutrition. This condition was more common in women than men (37% vs. 29%, *p* = 0.036).

### 3.2. Vitamin D Supplementation

A total of 26% (169/663) of the patients had some form of supplements containing vitamin D, either from prescribed drugs or from oral nutrition supplements, 10% in the vitamin D-insufficient group and 33% in the vitamin D-sufficient group. More women than men had supplements containing vitamin D (34% vs. 14%; *p* < 0.001). A significantly higher percentage of patients with sufficient levels of 25(OH)D had vitamin D-containing supplements (*p* = 0.001), (Table 1). The median difference in 25(OH)D between patients with and without supplements containing vitamin D was 27.3 nmol/L (83.2 vs. 55.9 nmol/L, (*p* < 0.001). The majority (85%) of supplements containing vitamin D were prescribed drugs, and the rest were oral nutrition supplements. Eight percent had both prescribed drugs and oral nutrition supplements containing vitamin D.

### 3.3. Vitamin D Status

**Figure 2:**
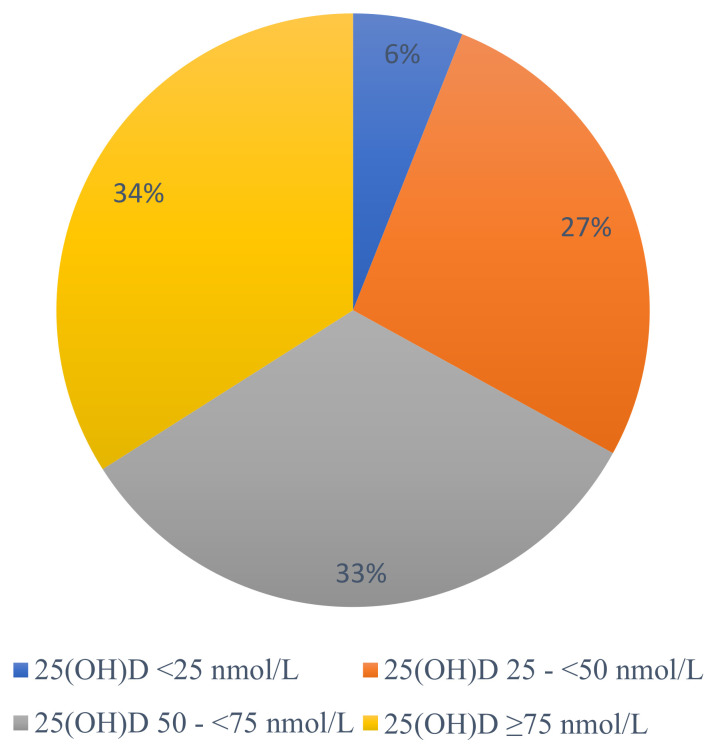
Distribution of vitamin D status. 25(OH)D, 25-Hydroxyvitamin D.

Median 25(OH)D in the total cohort was 62 nmol/L (43.5–83.1 nmol/L), ranging from 9–246 nmol/L. Thirty-three percent of patients had at least vitamin D insufficiency (25(OH)D <50 nmol/L), and 6% had vitamin D deficiency (25(OH)D <25 nmol/L). Sixty-seven percent had sufficient vitamin D levels, with 33% between 50 and <75 nmol/L and 34% >75 nmol/L (Figure 2). 

### 3.4. Seasonal Variations

Median 25(OH)D levels during the winter season (November to April) were significantly lower than during the summer season (May to October), 57.7 nmol/L compared to 64.3 nmol/L, *p* < 0.001. There was no statistical difference between the number of men and women visiting the COPD clinic during the winter season versus the summer season (*p* = 0.450).

### 3.5. Factors Associated with Vitamin D Insufficiency

**Figure 3:**
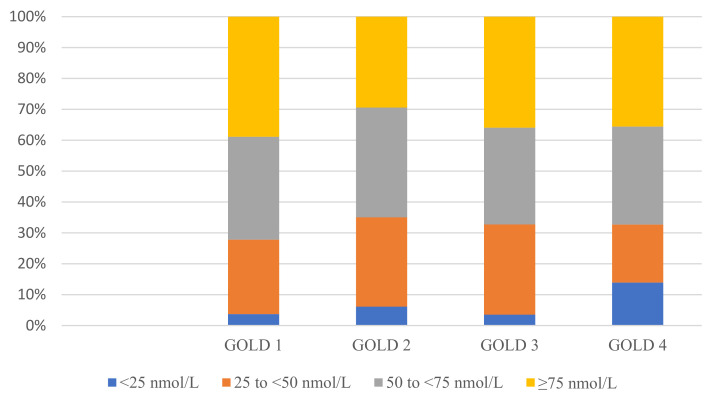
Vitamin D status (25-Hydroxyvitamin D, nmol/L) stratified by GOLD grade. Numbers within GOLD grade: GOLD grade 1 *n* = 54, GOLD grade 2, *n* = 228, GOLD grade 3 *n* = 284 and GOLD grade 4 *n* = 101. Abbreviations: GOLD, Global Initiative for Chronic Obstructive Lung Disease. Fisher-Freeman-Halton Exact test *p* = 0.031 (significant *p*-value < 0.05).

Table 1 shows the characteristics of subjects with and without vitamin D insufficiency. There were no differences in age, BMI, the severity of airflow obstruction, symptom measures or risk of malnutrition. Then again, patients with vitamin D insufficiency were proportionately more often men, were more often current smokers, had their 25(OH)D measured more often in the winter season and less often had supplements containing vitamin D. The proportions between the vitamin D status categories differ significantly in relation to GOLD grade (*p* = 0.031), (Figure 3). For example, among those with vitamin D deficiency, the proportions were 3.5–6% for GOLD grade 1–3 and 14% for GOLD grade 4. In the multivariable logistic regression analysis, gender, current smoking habits, lack of vitamin D-containing supplements and winter season for 25(OH)D blood sampling were independent predictors of vitamin D insufficiency (Table 2).

## 4. Discussion

In this real-life cohort of more than 600 patients, with mainly moderate to severe COPD, included at their first visit to a specialized secondary care COPD clinic, one in three patients had vitamin D insufficiency (25(OH)D less than 50 nmol/L) and 6% had vitamin D deficiency (25(OH)D less than 25 nmol/L), even though one in four had some form of vitamin D-containing supplement. Vitamin D insufficiency was independently determined by male gender, current smoking, lack of vitamin D-containing supplements and 25(OH)D blood sampling during the winter season. Age, BMI and FEV_1_ % predicted did not seem to be predictive variables in this cohort. Male patients more often had vitamin D insufficiency and less often vitamin D containing supplements.

In a previous study with a community-based cohort of elderly men in Sweden, the mean 25(OH)D was higher than in our cohort, 68.7 nmol/L, and less than 15% had vitamin D levels below 50 nmol/L [34]. This could reflect an association of vitamin D insufficiency with advanced COPD as shown previously [12,14], although within our secondary care COPD population, we did not find a specific association with severity of lung function impairment. Our findings are consistent with previous Scandinavian studies on fairly similar COPD cohorts [11,13] but in contrast to other, more recent, non-Scandinavian, studies [9,15]. In the study by Joliffe et al. [9] the mean serum level of 25(OH)D was lower (45.3 nmol/L) than in our study and other previous Scandinavian studies [11,13], and in the study by Burkes et al. [15] the number of participants was much greater (*n* = 1609), both of which are factors that might have affected the results. The median 25(OH)D level in our cohort was higher than in the study by Måhlin et al. in a similar cohort of patients with COPD in Sweden [13]. This difference may be due to the updated Nordic nutrition recommendations on vitamin D intake and the Swedish regulation on food fortification since then. The current recommendations regarding vitamin D intake are 10 µg daily up to 75 years of age and 20 µg daily for those above that age and other risk groups [25,35]. The food fortification regulation changed in 2018, to include more products and higher fortification levels [36]. It is therefore possible that more people nowadays cover their recommended intake of vitamin D from food.

No previous Scandinavian [11,13,37] nor more recent international [22,33] study on patients with COPD have found a significant association between gender and vitamin D status. In contrast to our findings Black et al. found a significantly higher level of 25(OH)D in men compared to women, 78.3 nmol/L versus 71.7 nmol/L, in a cohort from the Third National Health and nutrition Examination Survey (NHANES III) [18]. In our study, the higher prevalence of vitamin D insufficiency in men is probably related to the lower prevalence of men who take vitamin D-containing supplements. In total about one in four patients had some form of supplement containing vitamin D and 25(OH)D levels were significantly higher among them (83.2 nmol/L versus 55.9 nmol/L, *p* < 0,001). The reason for more prescription of vitamin D containing supplement in women is speculative but could potentially partly be explained by the higher prevalence of osteoporosis in elderly women which are commonly treated with calcium supplements in combination with vitamin D. Osteoporosis is common in patients with COPD [4,6] and a recent observational study showed that men were under-screened for osteoporosis [38]. It is therefore important to target the most common risk factors for osteoporosis with a personalized lifestyle approach. The focus should be on modifiable risk factors such as smoking cessation, physical activity, and nutritional interventions, including vitamin D supplementation in those who are insufficient [3,39,40].

The findings we present on determinants of vitamin D status regarding supplementation and seasonal variations are in agreement with previous findings in patients with COPD [9]. Kentson et al. found that ongoing vitamin D supplementation was the most important intervention to maintain vitamin D levels above 50 nmol/L, in a cohort of patients with severe COPD [37]. Season was a strong predictor for vitamin D insufficiency in our cohort. A greater proportion of participants with vitamin D insufficiency had their first visits during November to April and the median 25(OH)D levels were significantly lower then, compared to the summer season. Seasonal variations in vitamin D status are well known, and several studies have shown the same association in patients with COPD [5,9,12]. Since vitamin D is synthesized in the skin under the influence of UVB radiation [24], and the sun exposure is insufficient during winter in the northern latitudes [25], vitamin D supplementation may be needed to an even greater extent, certainly in patients with COPD, who due to their condition are less physically active [41] and therefore potentially less exposed to sunlight. Physical activity is therefore an important part of pulmonary rehabilitation [1]. Current smoking was a significant determinant for vitamin D insufficiency in our cohort, and similar results have been found in previous studies on patients with COPD [12,15]. Smoking cessation is essential for the prevention and treatment of COPD, as well as nutritional interventions, including dietary supplements [39,42].

### Strengths and Limitations

The main strengths of this study are the relatively large number of patients and the representativity of the COPD cohort, as data were collected from the patients first visit to a specialized secondary care COPD clinic, with a considerable a range of disease severity, and visits across all seasons – all of which are factors that increase the generalizability of our results.

Our study also has some limitations. One limitation is that the instrument and analysis method for 25(OH)D changed between the two periods of data collection. However, it is not uncommon in clinical practice for reference values or analysis methods to change over time. Since there is only one single measurement for 25(OH)D and no change in reference values, the potential difference between the serum and plasma samples has probably not affected the categorization of participants into the vitamin D-sufficient versus the vitamin D-insufficient group. The absolute bias for plasma was 3%, according to the laboratory (personal communication). Another limitation is that we are lacking data on comorbidities and the use of other medications that could potentially be associated with vitamin D insufficiency. Indeed, the presence of osteoporosis might have affected the number of patients who had supplementation containing vitamin D, as well as the use of corticosteroids might affect vitamin D status [23]. A further limitation is that we do not know how many of the patients, with documentation of supplements containing vitamin D, actually had vitamin D supplementation prescribed to treat vitamin D deficiency. We are also lacking data on dietary intake, which would have been useful for the assessment of the intake of vitamin D from food and drinks.

## 5. Conclusions

In conclusion, our results add to previous findings that vitamin D insufficiency is common in patients with COPD. Our study also highlights lower levels of vitamin D and a lower prevalence of vitamin D-containing supplements in men, which, to our knowledge, has not been previously reported. Our findings can help clinicians to identify patients who are at risk of vitamin D insufficiency and allow correction with supplementation where appropriate—regardless of gender at northern latitudes.

## Figures and Tables

**Table 1:** Patient characteristics.

	Total	Women	Men	*p*-Value	Vitamin D Insufficiency	Vitamin D Sufficiency	*p*-Value
**Patients, *n*** (%)	667	394 (59)	273 (41)		221 (33)	446 (67)	
**Sex, *n*** (%)							
**Male**	273 (41)				113 (51)	160 (36)	< 0.001 *
**Age,** median (Q1–Q3)	73 (66–79)	73 (67–79)	72 (66–78)	0.520 ^2^	73 (66–79)	73 (67–79)	0.757 ^2^
**BMI, kg/m^2^,** median (Q1–Q3)	25.7 (21.9–30)	25.6 (21.4–30.4)	26 (22.5–29.4)	0.306 ^2^	26 (22.6–30.7)	25.5 (21.6–29.8)	0.118 ^2^
**Current smoking, *n*** (%)	189 (28)	107 (57)	82 (43)	0.395 ^1^	73 (33)	116 (26)	0.055 ^1^
**FEV_1_ % predicted, *n*** = 632							
median (Q1–Q3)	47 (36–62)	47 (36–61)	49 (35–63)	0.569 ^2^	48 (36–60)	47 (36–62)	0.866 ^2^
**GOLD grade, *n*** (%)							
**1**	54 (8)	28 (7)	26 (10)	0.439 ^1^	15 (7)	39 (9)	0.772 ^1^
**2**	228 (34)	134 (34)	94 (34)		80 (36)	148 (33)	
**3**	284 (43)	176 (45)	108 (40)		93 (42)	191 (43)	
**4**	101 (15)	56 (14)	45 (16)		33 (15)	68 (15)	
**CAT score,**							
median (Q1–Q3)	20 (14–25)	21 (15–25)	19 (14–25)	0.085 ^2^	21 (15–26)	20 (14–25)	0.096 ^2^
**Risk of malnutrition, *n*** (%)	209 (34)	136 (37)	73 (29)	0.036 ^1^	61 (30)	148 (36)	0.143 ^1^
**25(OH)D, nmol/L,**							
median (Q1–Q3)	62 (43.5–83.1)	65.3 (47.3–87.6)	55 (40.7–77.7)	< 0.001 ^2^*	36.7 (29.7–43.4)	75 (62–94.3)	< 0.001 ^2^*
**Documented vitamin D**							
**supplementation, *n*** (%)	169 (26)	132 (34)	37 (14)	< 0.001 ^1^*	22 (10)	147 (33)	< 0.001 ^1^*
**Season, *n*** (%)							
**Summer**	345 (52)	199 (58)	146 (42)	0.450 ^1^	91 (41)	254 (57)	< 0.001 ^1^*
**Winter**	322 (48)	195 (61)	127 (39)		130 (59)	192 (43)	

All values represent number (%) or median (quartiles (Q) 1 and 3). ^1^ Pearson Chi-Square. ^2^ Independent Samples Mann-Whitney U Test. * Significant *p*-value, <0.05. BMI, Body mass index. FEV_1_ % predicted, Forced expiratory volume in one second in percent of predicted value. GOLD, Global Initiative for Chronic Obstructive Lung disease. CAT, COPD assessment test. 25(OH)D, 25-Hydroxyvitamin D.

**Table 2:** Result from the multivariable logistic regression analysis, showing determinants of vitamin D insufficiency among patients with COPD.

Coefficient	OR	95% CI	*p*-Value
**Age**	1.01	0.99–1.03	0.201
**Gender**	1.64	1.14–2.35	0.007 *
**BMI**	1.01	0.98–1.04	0.448
**FEV_1_ % predicted**	1.00	0.99–1.01	0.570
**Smoking**	1.53	1.02–2.31	0.040 *
**Season**	2.08	1.45–2.42	< 0.001 *
**Supplementation containing vitamin D**	4.11	2.42–6.98	< 0.001 *
*n*= 623			
**Nagelkerke’s R^2^ = 0.152**			

Abbreviations: COPD, Chronic Obstructive Pulmonary Disease. OR, odds ratio. CI, confidence interval. * Significant *p*-value < 0.05. BMI, Body mass index. FEV_1_ % predicted, Forced expiratory volume in one second in percent of predicted value. *n* = total number. Coding: 25 Hydroxyvitamin D, >50 nmol/L 0 and <50 nmol/L 1 gender, female 0 and male 1, smoking, former and never-smoker 0 and current 1, documented vitamin D supplementation, yes 0 and no 1 and season, summer season 0 and winter season 1. Age, BMI and FEV_1_ % predicted are continuous variables.

## Data Availability

The data that supports the findings of this study are available upon reasonable request from the corresponding author (M.M.). The data are not publicly available due to local legislation related to data derived from patients’ electronic medical records.
